# Optimization of PET acquisition time for thoracic lesions in ^18^F-FDG PET/MRI: a retrospective analysis

**DOI:** 10.1186/s41747-026-00787-y

**Published:** 2026-10-07

**Authors:** Haifeng Gu, Yue Jiang, Jun Cai, Gang Yang, Jianrui Li, Sui Chen, Song Luo, Longjiang Zhang

**Affiliations:** 1 Department of Diagnostic Radiology, Jinling Hospital, Affiliated Hospital of Medical School, Nanjing University, Nanjing, China; 2 Department of Radiology, Geriatric Hospital of Nanjing Medical University, Nanjing, China

**Keywords:** Fluorodeoxyglucose F18, Patient comfort, Magnetic resonance imaging, Positron emission tomography, Thorax

## Abstract

**Objective:**

This study aimed to define the minimum positron emission tomography (PET) acquisition time for chest PET/magnetic resonance imaging (MRI), providing image quality and lesion detectability comparable to a 20-min reference.

**Materials and methods:**

In this retrospective study, list-mode data from 101 patients undergoing chest ^18^F-fluorodeoxyglucose (^18^F-FDG) PET/MRI on a 3-T PET/MRI system were reconstructed to simulate 10 acquisition times (0.5–20 min). Two readers assessed subjective image quality using a 5-point Likert scale. Quantitative parameters, including maximum, mean of the lesion standardized uptake value (L_SUV_max_, L_SUV_mean_), image noise ratio (INR), and lesion-to-background ratio (LBR), were measured. Lesion detectability was defined as the miss rate relative to lesions visible on the 20-min reference acquisition.

**Results:**

In 101 patients, 283 lesions were identified. Subjective scores increased from a median of 2 (interquartile range 2–2) at 0.5 min to 5 (5–5) at 7 min (*p* < 0.001), then plateaued (7–20 min; all *p* = 1.000). L_SUV_max_, L_SUV_mean_, and LBR inversely correlated with scan duration (all *p* < 0.001). L_SUV_mean_ stabilized at 3 min (*p* = 1.000), while L_SUV_max_/LBR stabilized at 5 min (*p* = 1.000). INR required 10 min (*p* ≥ 0.054). Lesion miss rates increased time dependently, from 0% at 15 min to 7.77% at 0.5 min.

**Conclusion:**

A 7-min acquisition time provided subjective quality and detectability comparable to the 20-min protocol, supporting routine use. For quantitative assessments, 3–5 min sufficed, and patients with shorter ^18^F-FDG uptake or larger lesions may be adequately assessed with 2 min.

**Key Points:**

***Question*** What is the minimum PET acquisition time for chest 18F-FDG PET/MRI that maintains image quality and lesion detectability equivalent to a 20‑min reference?

***Findings*** A 7‑min PET acquisition matches 20‑min image quality and detectability; quantitative parameters stabilize at 3‑5 min; 2 min suffices for patients with shorter uptake or larger lesions.

***Relevance statement*** Reducing PET acquisition time to 7 min enhances patient comfort, reduces motion artifacts, and increases scanner throughput without compromising chest PET/MRI diagnostic performance, thereby improving patient experience and workflow efficiency. Further clinical validation is needed.

**Keywords:**

Fluorodeoxyglucose F18, Magnetic resonance imaging, Patient comfort, Positron emission tomography, Thorax

**Graphical Abstract:**

**Figure:**
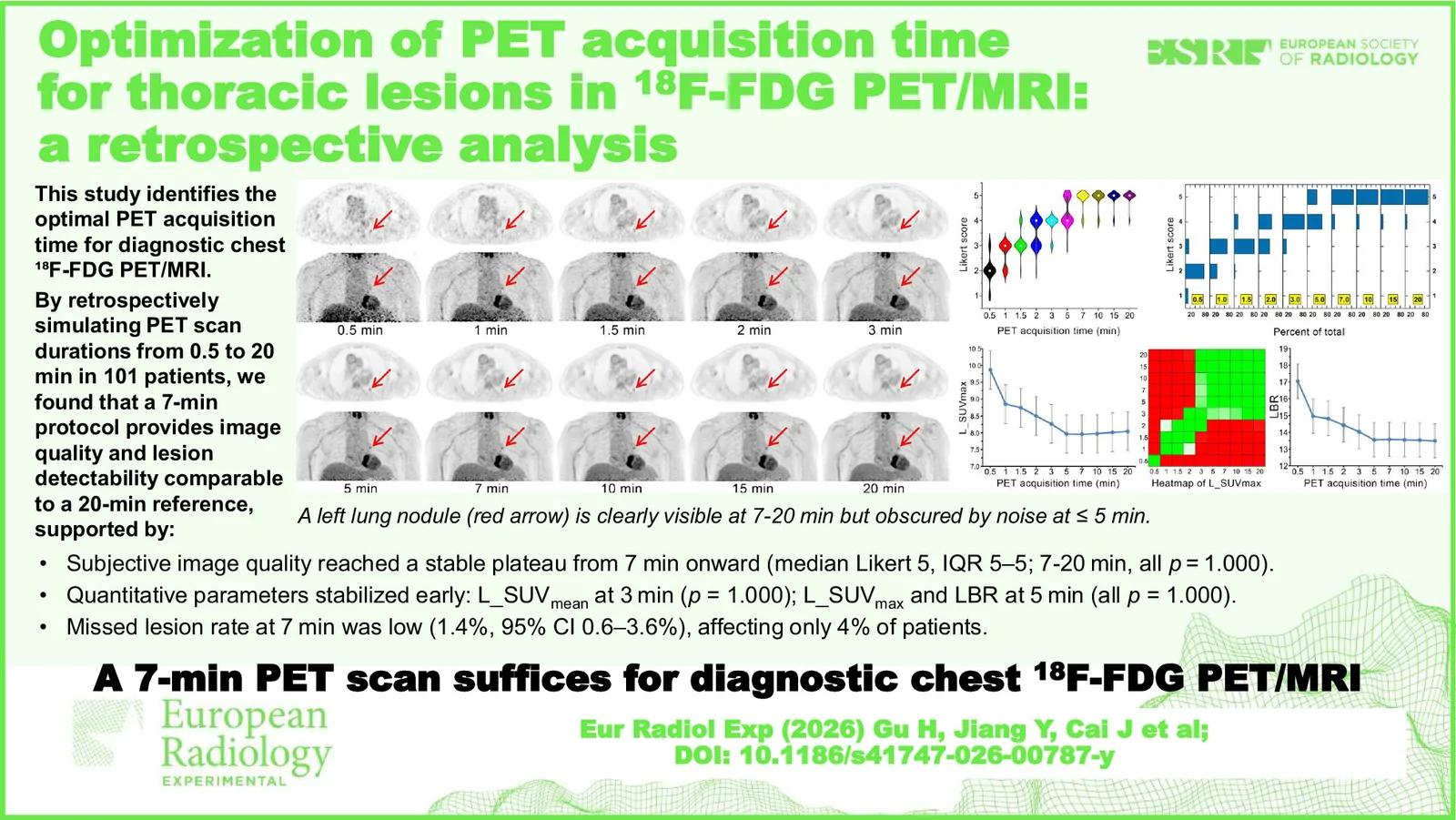

## Background

The incidence of thoracic diseases, particularly lung cancer, has been increasing [1]. Computed tomography (CT) is widely used for lesion detection but has limited specificity, necessitating a more robust diagnostic approach. Given the relatively low specificity in lung lesion based on CT morphological standards, there has been an urgent demand for a more robust diagnostic standard. Positron emission tomography (PET)/CT serves as a valuable tool for further precise differential diagnosis, confirming the presence of lymph node metastases and effectively distinguishing between benign and malignant chest lesions [2]. However, PET/CT has limited sensitivity for subtle thoracic lesions [3] and exposes patients to ionizing radiation [4].

Magnetic resonance imaging (MRI) provides a radiation-free alternative for thoracic imaging [5, 6]. However, it cannot capture metabolic activity, limiting its ability to differentiate benign from malignant lesions. PET/MRI overcomes this limitation by adding metabolic information while avoiding CT-related radiation [7].

The integrated PET/MRI systems enable simultaneous data by integrating functional information from PET and anatomic/functional information from MRI, which results in more precise image fusion [8]. Due to the inherently slower nature of MRI, PET scan durations can be correspondingly prolonged. It has been established that extending the PET acquisition time per bed enhances image quality and signal-to-noise ratio (SNR) [9, 10]. However, this benefit is counterbalanced by the drawback of patient tolerance, as overly long protocols risk discomfort and motion artifacts, which can compromise image quality. A solution is to optimize scan protocols by either reducing PET acquisition time or streamlining MRI sequences to better balance the duration of both modalities. For example, a recent study by Lütje et al demonstrated that the optimal acquisition time of ^68^Ga-prostate-specific membrane antigen (PSMA)-ligand PET/MRI in patients with prostate cancer was identified to be 4 min per bed position, balancing imaging quality and lesion detectability [11]. Meanwhile, Noto et al reported that reduction of acquisition duration (if < 3 min per bed position) results in an increase in likelihood of halo artifacts [12]. To date, given the limited existing research on this topic and the relative scarcity of studies on ^18^F-fluorodeoxyglucose (^18^F-FDG) PET/MRI for thoracic examinations, it is crucial to evaluate the impact of different PET acquisition times on the quality of thoracic PET images.

In this study, we used the 20-min acquisition as a reference and retrospectively reconstructed shorter acquisition times, enabling systematic evaluation of image quality and lesion detectability. Accordingly, the purpose of this study was to identify the shortest PET acquisition time that is non-inferior to the 20-min reference for image quality and lesion detectability in thoracic ¹⁸F-FDG PET/MRI, thereby enabling workflow optimization.

## Materials and methods

### Patients

The study followed institutional guidelines and was approved by the local Ethics Committee (2025DZKY-047-01). As it was a retrospective study, written informed consent from participants was waived. We reviewed 126 patients retrospectively who underwent ^18^F-FDG Chest PET/MRI between March 2022 and April 2023. Exclusion criteria included contraindications to MRI/gadolinium, lack of ^18^F-FDG-avid chest lesions, recent antitumor therapy, substantial artifacts, scan duration < 20 min, or incomplete datasets.

### Chest PET/MRI imaging protocol

Prior to PET/MRI examinations, all patients were required to fast for at least 6 h, maintain fasting blood glucose levels < 8.0 mmol/L, and refrain from vigorous physical activity. An appropriate dose of ^18^F-FDG based on the patient’s body weight was intravenously administered before imaging, with a mean activity of 4.11 ± 0.91 MBq/kg (range 2.19–5.88 MBq/kg). Following a standardized tracer uptake period of 66.31 ± 12.21 min, patients underwent PET/MRI acquisition in supine position with arms down by an integrated 3.0-T time-of-flight PET/MRI system (Signa, GE Healthcare) [13].

First, a rapid whole-body PET/MRI acquisition was performed, spanning from the vertex of the skull to mid-thigh (4–5 bed positions, based on the patient’s height, 2 min per bed). Subsequently, a dedicated chest PET/MRI acquisition was conducted at a single bed, with the scanning position determined based on the whole-body PET/MRI. Considering the time required for routine chest MRI protocols, the PET acquisition time was set at 20 min to match the total MRI duration (approximately 20 min; see Table 1). This ensured complete temporal synchronization between modalities. PET data were acquired as list-mode files, allowing for retrospective reconstruction of various acquisition durations. Attenuation correction was performed using a Dixon-based volumetric interpolated breath-hold examination sequence. As the study was restricted to chest PET/MRI, the whole-body PET/MRI findings were excluded from analysis.

**Table 1:** Routine chest MRI protocol performed simultaneously with PET acquisition

Sequence	Orientation	TR (ms)	TE (ms)	Slice thickness (mm)	Matrix	Acquisition time
T2WI FRFSE	Axial	8,521	83	4.0	256 × 352	9:44
DWI (b = 600 s/mm²)	Axial	8,000	58.3	4.0	128 × 128	4:16
LAVA	Axial	4.7	1.1	2.0	180 × 288	2:26
T2WI PROPELLER	Coronal	8,571	117.3	4.0	352 × 352	3:09
Total						19:35

*DWI* Diffusion-weighted imaging, *FRFSE* Fast recovery fast spin-echo, *LAVA* Liver acquisition with volume acceleration, *PROPELLER* Periodically rotated overlapping parallel lines with enhanced reconstruction, *T2WI* T2-weighted imaging, *TE* Echo time, *TR* Repetition time

### PET imaging reconstruction

In order to determine the optimal PET acquisition time, we retrospectively reconstructed the raw 20-min PET list-mode data from all patients with 10 simulated time intervals emulating reduced PET acquisition times [14]. Finally, 10 groups of data with different PET acquisition time intervals (0.5, 1, 1.5, 2, 3, 5, 7, 10, 15, and 20 min) were obtained. Reconstructions used time-of-flight, point spread function correction, ordered subset expectation maximization (2 iterations, 28 subsets), 5.0 mm Gaussian filter, 600 × 250 mm FOV, 256 × 256 matrix, and 2.8 mm slice thickness.

### Image analysis: subjective image quality evaluation

Two readers (16 and 14 years of experience) independently assessed images in random order using PET Volume Computed Assisted Reading (PET VCAR; GE Healthcare) software, blinded to acquisition parameters and clinical information. Disagreements were resolved by consensus.

A 5-point Likert scale was employed for assessment [15]. 1 = non-diagnostic (unable to discriminate lesions from background); 2 = limited (slightly discriminate lesions from background); 3 = acceptable (ability to discriminate lesions with significant image noise); 4 = good (ability to discriminate lesions with low image noise); 5 = excellent (ability to discriminate lesions with no image noise).

### Image analysis: objective image quality evaluation

Following the above subjective evaluation of image quality review, one investigator (the nuclear medicine physician) reviewed each patient’s PET images in conjunction with their medical history and all other available records to identify lesions. According to the principles of PET imaging, a lesion was deemed to be present in an area of abnormal radionuclide concentration (excluding normal physiological uptake).

For each patient’s different PET image acquisition time sets, we assessed the following parameters:

i. SUV related parameters: maximum value of the lesion (L_SUV_max_); mean value of the lesion (L_SUV_mean_); standard deviation of the lesion (L_SUV_sd_); mean value of the background (B_SUV_mean_); standard deviation of the background (B_SUV_sd_);
ii. signal-to-noise ratio of the lesion (L_SNR);
iii. image noise ratio (INR) of the background;
iv. lesion-to-background ratio (LBR);
v. metabolic tumor volume (MTV).

The PET images were analyzed starting from 20 min down to 0.5 min to avoid bias in lesion detectability. All measurements were performed by PET VCAR. For each lesion, a volume of interest was defined on the 20-min reference image using the iterative adaptive algorithm [16] and verified by the reader. This volume of interest was then propagated to all other reconstructed time points for the same patient, and L_SUV_max_, L_SUV_mean_, L_SUV_sd_, and MTV were automatically recorded from each time point. For background measurement, a circular region of interest of approximately 2–3 cm in diameter was manually placed in the lumen of the ascending aorta (avoiding wall and calcification) at the level of the tracheal bifurcation on the 20-min reference image [17, 18]. This region of interest was then copied to the identical positions in all other reconstructed time points, and B_SUV_mean_ and B_SUV_sd_ were recorded accordingly. Finally, L_SNR, INR, and LBR of each group of PET acquisition time were calculated as follows [19, 20]:

$$\begin{array}{ccc}L{\rm{\_}}\_{SNR}=\frac{{L\_{\rm{\_}}{SUV}}_{{mean}}}{{L{\rm{\_}}\_{SUV}}_{{sd}}}, & {IN}=\frac{{B{\rm{\_}}\_{SUV}}_{{sd}}}{{B{\rm{\_}}\_{SUV}}_{{mean}}}, & {LBR}=\frac{{L\_{\rm{\_}}{SUV}}_{\max }}{{B{\rm{\_}}\_{SUV}}_{{mean}}}\end{array}$$

### Lesion detectability evaluation

Lesion detectability was evaluated by comparing each simulated acquisition time to the 20-min reference. For each patient, the total number of lesions visible on the 20-min images was first recorded by two readers in consensus. For each of the other nine time points, the number of lesions that were missed (*i.e*., not identified) was recorded. The miss rate was calculated as the proportion of lesions visible on the 20-min reference that were missed at each shorter acquisition time.

### Subgroup analysis

To further evaluate the robustness of the findings across different clinical scenarios, subgroup analyses were performed stratified by ^18^F-FDG uptake period and by lesion size. ^18^F-FDG uptake period was categorized into two groups (≤ 80 min and > 80 min) based on the distribution of uptake times. Lesions were divided into three size groups (< 15 mm, 15–20 mm, > 20 mm) based on MTV-derived equivalent spherical diameter.

### Statistical analysis

All statistical analyses were conducted using IBM SPSS Statistics 27 software (IBM Corp.). Subjective image quality scores were summarized as median (interquartile range, IQR). Interobserver agreement was assessed using weighted Cohen κ with quadratic weights [21]. Subjective scores were compared using Friedman’s test with Bonferroni-corrected Wilcoxon *post hoc* tests. Objective metrics were analyzed using linear mixed-effects models (acquisition time as fixed effect, random intercepts for patients and lesions), with Bonferroni correction for pairwise comparisons. Subgroup analyses used the same model structure. The *p*-values lower than 0.05 were considered to be statistically significant.

## Results

### Patient population

**Fig. 1:**
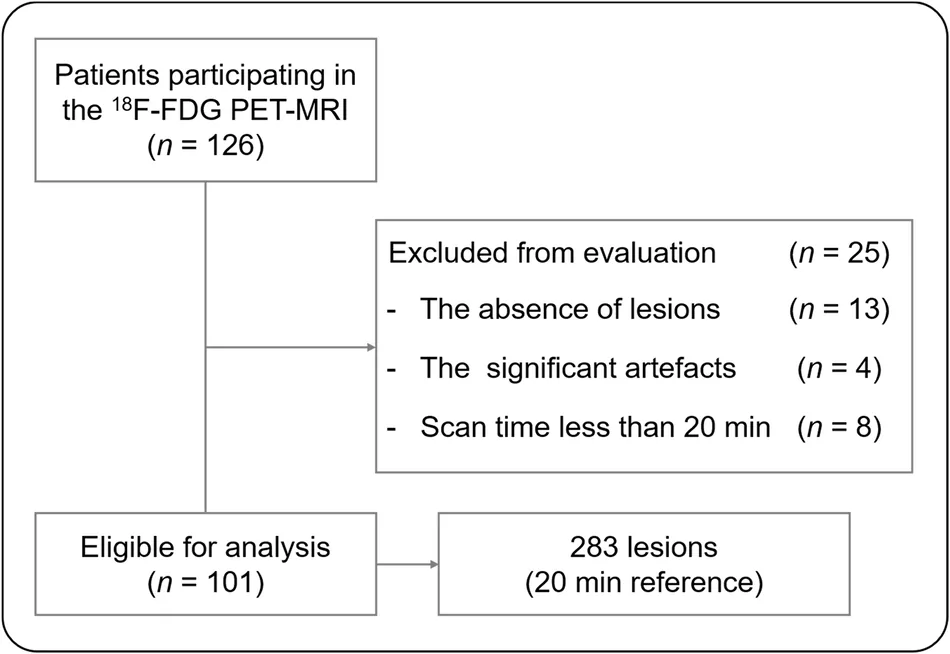
Flowchart illustrating the patient enrollment process

After exclusion of 25 patients (Fig. 1), a total of 101 patients (77 males, 24 females; average age 63.8 ± 9.6 years; range 39–84 years) were included in the final analysis. Of these, 89 (88.1%) were pathologically confirmed. The most common primary malignancy was lung cancer (*n* = 80), followed by esophageal cancer (*n* = 15). Detailed patient and lesion characteristics are provided in Supplementary Table S1. A total of 283 lesions were identified on the 20-min reference images (1–6 lesions per patient).

### Subjective evaluation of PET image quality

Table 2 summarizes the two readers’ evaluations of subjective image quality for all 101 patients included. As PET acquisition time increased from 0.5 to 20 min, both readers consistently gave higher subjective image quality ratings (all *p* < 0.001), with interobserver agreement improving from substantial (κ: 0.676–0.772 at 0.5–10 min) to almost perfect (κ: 0.826–0.864 at 15–20 min).

**Table 2:** Subjective image quality scores by PET acquisition time

PET acquisition time (min)	Reader 1	Reader 2	Weighted kappa (95% CI)	Both readers
0.5	2 (2–2)	2 (2–2)	0.676 (0.541–0.812)	2 (2–2)
1	3 (2–3)	3 (2–3)	0.636 (0.496–0.775)	3 (2–3)
1.5	3 (3–3)	3 (3–3)	0.667 (0.494–0.841)	3 (3–3)
2	4 (3–4)	4 (3–4)	0.761 (0.634–0.889)	4 (3–4)
3	4 (4–4)	4 (4–4)	0.694 (0.537–0.851)	4 (4–4)
5	4 (4–5)	4 (4–5)	0.772 (0.647–0.898)	4 (4–5)
7	5 (5–5)	5 (5–5)	0.751 (0.562–0.941)	5 (5–5)
10	5 (5–5)	5 (5–5)	0.772 (0.580–0.964)	5 (5–5)
15	5 (5–5)	5 (5–5)	0.826 (0.634–1.018)	5 (5–5)
20	5 (5–5)	5 (5–5)	0.864 (0.680–1.049)	5 (5–5)
*p*-value	< 0.001	< 0.001		< 0.001

Data are presented as median (interquartile range, Q1–Q3). Weighted κ values (with 95% confidence intervals) were calculated with quadratic weights, *p*-values from Friedman's test for overall comparison across acquisition times

*PET* Positron emission tomography

**Fig. 2:**
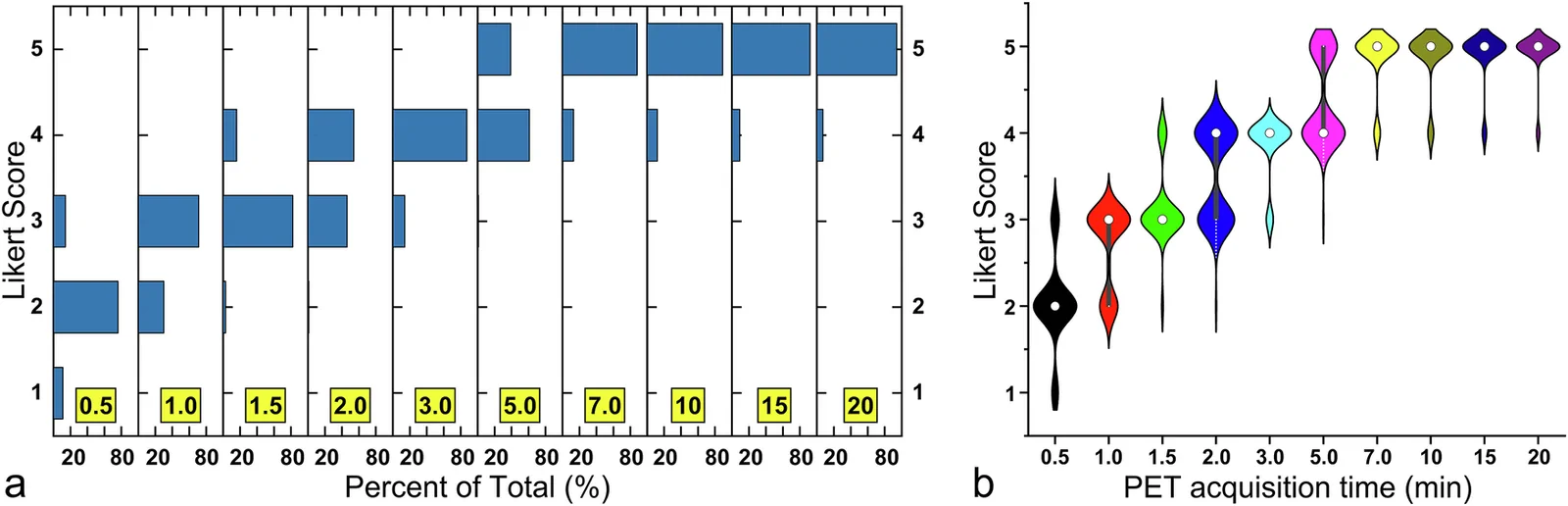
Distribution of consensus Likert scores across PET acquisition times. **a** Percentage stacked bars show the proportion of each score. **b** Violin plots display the full distribution, median (white dot), and interquartile range (thick bar). Scores improve with longer acquisition times and plateau at 7 min, with no significant differences among 7–20 min (all *p* = 1.000, Friedman test with Bonferroni correction)

**Fig. 3:**
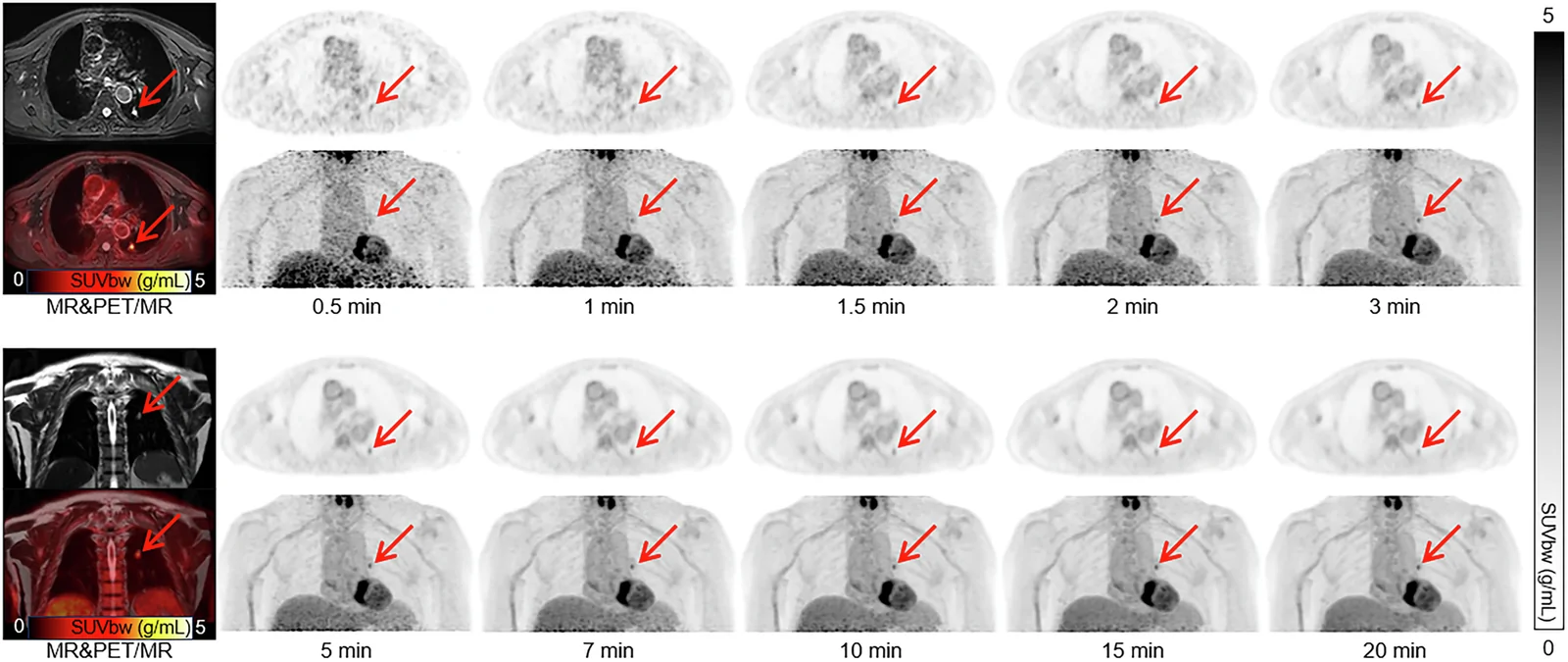
Representative PET images across acquisition times in a lung cancer patient. Axial PET, MR, PET/MRI fusion, and coronal maximum intensity projection (MIP) PET images of a 71‑year‑old man with lung cancer. PET images were acquired at 0.5–20 min. A small nodule (red arrow) with high ^18^F-FDG uptake in the lower lobe of the left lung is clearly visible from 7 to 20 min, faintly visible at 1.5–5 min, and almost invisible at 0.5–1 min. SUV_bw_, Standard uptake value normalized for patient body weight

Consensus scores increased from median 2 (IQR 2–2) at 0.5 min to 5 (IQR 5–5) at 20 min (*p* < 0.001). Scores improved significantly up to 7 min (*e.g*., 5 min *versus* 7 min: *p* = 0.011), with no differences among 7–20 min (all *p* = 1.000, Fig. 2). Figure 3 shows MRI, PET, and fusion images of one patient and illustrates how image quality and lesion detectability increase as acquisition time increases.

### Objective metrics of PET image quality

**Fig. 4:**
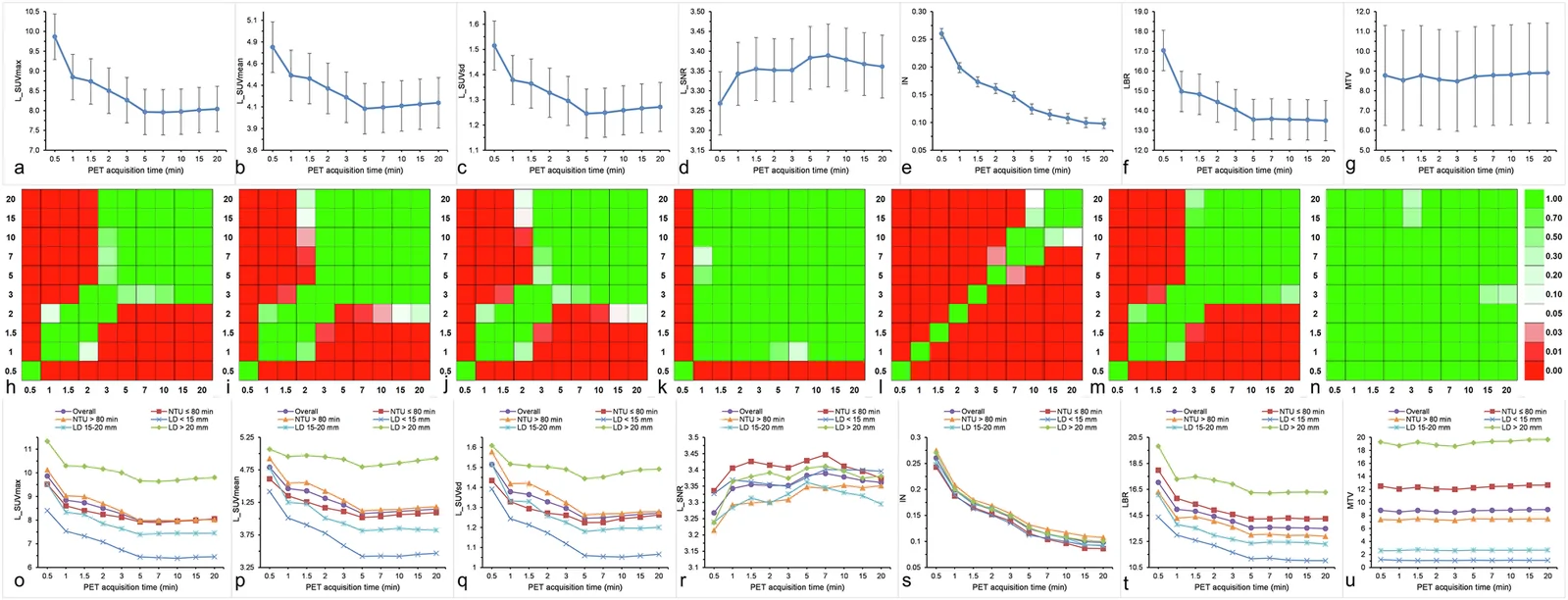
Quantitative PET parameters across PET acquisition times: marginal means, heatmaps, and subgroup analyses. **a**–**g** Estimated marginal means (error bars: 95% CI) of the seven PET parameters *versus* PET acquisition time. **h**–**n** Heatmaps of Bonferroni-corrected pairwise *p*-values. Red shades indicate significant differences (*p* < 0.05), green shades indicate non-significant differences (*p* ≥ 0.05). Solid green blocks indicate stable plateaus (non-significant consecutive pairwise comparisons). **o**–**u** Overlaid marginal means for the overall cohort, ^18^F-FDG uptake subgroups (≤ 80 min, > 80 min), and lesion size subgroups (< 15 mm, 15–20 mm, > 20 mm). Line styles distinguish subgroups (see legend); error bars omitted. For all rows, panels correspond to: (**a**, **h**, **o**) L_SUV_max_, (**b**, **i**, **p**) L_SUV_mean_, (**c**, **j**, **q**) L_SUV_sd_, (**d**, **k**, **r**) L_SNR, (**e**, **l**, **s**) INR, (**f**, **m**, **t**) LBR, (**g**, **n**, **u**) MTV. L_SUV_max_, Maximum standardized uptake value of the lesion; L_SUV_mean_, Mean standardized uptake value of the lesion; L_SUV_sd_, Standard deviation of standardized uptake value within the lesion; L_SNR, Lesion signal-to-noise ratio; INR, Image noise ratio; LBR, Lesion-to-background ratio; MTV, Metabolic tumor volume (cm³)

The estimated marginal means with 95% confidence intervals (CI) for all quantitative metrics across the 10 acquisition times are presented in Table 3 and visualized in Fig. 4a–g. The corresponding pairwise comparisons with Bonferroni correction are shown as heatmaps in Fig. 4h–n.

**Table 3:** Estimated marginal means of quantitative metrics by PET acquisition time

PET acquisition time (min)	L_SUV_max_	L_SUV_mean_	L_SUV_sd_	L_SNR	IN	LBR	MTV
0.5	9.87 (9.29–10.44)	4.79 (4.50–5.08)	1.52 (1.42–1.61)	3.27 (3.19–3.35)	0.26 (0.25–0.27)	17.04 (16.02–18.06)	8.78 (6.25–11.30)
1	8.85 (8.27–9.42)	4.46 (4.17–4.75)	1.38 (1.28–1.48)	3.34 (3.26–3.42)	0.20 (0.19–0.21)	14.97 (13.95–15.99)	8.53 (6.01–11.06)
1.5	8.74 (8.16–9.31)	4.43 (4.14–4.72)	1.36 (1.27–1.46)	3.36 (3.28–3.43)	0.17 (0.16–0.18)	14.82 (13.80–15.84)	8.77 (6.24–11.29)
2	8.50 (7.93–9.08)	4.31 (4.02–4.60)	1.33 (1.23–1.43)	3.35 (3.27–3.43)	0.16 (0.15–0.17)	14.43 (13.42–15.45)	8.57 (6.04–11.09)
3	8.26 (7.69–8.84)	4.21 (3.92–4.50)	1.30 (1.20–1.39)	3.35 (3.27–3.43)	0.15 (0.14–0.16)	14.04 (13.02–15.06)	8.48 (5.95–11.00)
5	7.97 (7.39–8.54)	4.08 (3.79–4.37)	1.25 (1.15–1.34)	3.38 (3.30–3.46)	0.12 (0.12–0.13)	13.55 (12.53–14.57)	8.72 (6.20–11.25)
7	7.96 (7.39–8.53)	4.09 (3.80–4.39)	1.25 (1.15–1.35)	3.39 (3.31–3.47)	0.11 (0.11–0.12)	13.58 (12.56–14.60)	8.78 (6.26–11.31)
10	7.97 (7.40–8.55)	4.11 (3.82–4.40)	1.26 (1.16–1.36)	3.38 (3.30–3.46)	0.11 (0.10–0.11)	13.55 (12.53–14.57)	8.81 (6.28–11.33)
15	8.01 (7.44–8.59)	4.13 (3.84–4.42)	1.27 (1.17–1.36)	3.37 (3.29–3.45)	0.10 (0.10–0.11)	13.53 (12.52–14.55)	8.89 (6.36–11.41)
20	8.04 (7.47–8.61)	4.15 (3.86–4.44)	1.27 (1.17–1.37)	3.36 (3.28–3.44)	0.10 (0.10–0.11)	13.49 (12.47–14.51)	8.90 (6.38–11.43)
*p*-value	< 0.001	< 0.001	< 0.001	< 0.001	< 0.001	< 0.001	0.081

Data are presented as estimated marginal means (95% confidence intervals). All metrics were analyzed using linear mixed-effects models with acquisition time as a fixed effect and random intercepts for patients and for lesions nested; *p-*values from the overall comparison across acquisition times

*L_SUV*_*max*_ Maximum standardized uptake value of the lesion, *L_SUV*_*mean*_ Mean standardized uptake value of the lesion, *L_SUV*_*sd*_ Standard deviation of standardized uptake value within the lesion, *L_SNR* Lesion signal-to-noise ratio, *INR* Image noise ratio, *LBR* Lesion-to-background ratio, *MTV* Metabolic tumor volume (cm³)

L_SUV_max_, L_SUV_mean_, L_SUV_sd_, and LBR decreased with time (all *p* < 0.001). All pairwise comparisons from 3 min were non-significant (*p* ≥ 0.377). L_SUV_mean_ reached complete stability (*p* = 1.000) at 3 min, while the others did so at 5 min.

INR decreased significantly with longer acquisition times (*p* < 0.001). From 10 min onward, all pairwise comparisons among time points were non-significant (all *p* ≥ 0.054).

L_SNR showed no significant differences in any pairwise comparisons among time points from 1 to 20 min (all *p* = 1.000), except for those involving 0.5 min, which were all significant (*p* ≤ 0.007). MTV showed no significant differences in any pairwise comparisons across all acquisition times (all *p* ≥ 0.340).

Across subgroups, the same quantitative trends are visualized in Fig. 4o–u, which overlays the marginal means for the overall cohort, ^18^F-FDG uptake subgroups (≤ 80 min, > 80 min), and lesion size subgroups (< 15 mm, 15–20 mm, > 20 mm). Line styles distinguish subgroups (error bars omitted).

### Lesion identification and missed detection rate

**Fig. 5:**
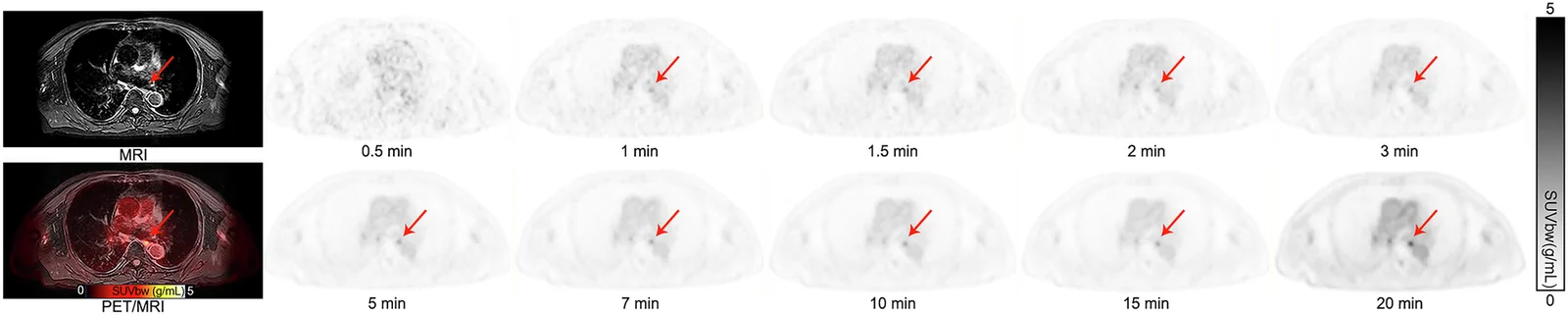
PET images of a missed lesion due to short acquisition time in a patient with esophageal cancer. Axial PET, MR, and PET/MRI fusion images of a 63‑year‑old man with squamous cell carcinoma of the esophageal inlet. PET images were acquired at 0.5–20 min. The lesion (red arrow) is clearly visible from 7 to 20 min, faintly visible at 3–5 min, almost invisible at 1–2 min, and not detectable at 0.5 min. SUV_bw_, Standard uptake value normalized for patient body weight

Using the 20-min images as reference, a total of 283 lesions were identified in the 101 patients. Compared with this reference, the lesion miss rate increased with shorter acquisition times, from 0.00% at 15 min to 7.77% (22/283) at 0.5 min. A representative case of a missed lesion at short acquisition times is shown in Fig. 5. The 95% CI for the miss rates and patient‑level statistics are also summarized in Table 4. Detailed characteristics of lesions, including lesion size, SUV_max_, anatomical location, and first detectable time, are provided in Supplementary Table S2. The relationship between lesion size and the shortest acquisition time at which each lesion was first detected is shown in Supplementary Fig. S1.

**Table 4:** Lesion miss rates and patient-level statistics at each acquisition time

PET acquisition time (min)	Missed lesions	Miss rate (%)	95% CI	Patients with ≥ 1 missed lesion	Patients with ≥ 2 missed lesions
0.5	22	7.77	(5.19–11.49)	21 (20.79%)	1 (0.99%)
1	10	3.53	(1.93–6.38)	9 (8.91%)	1 (0.99%)
1.5	9	3.18	(1.68–5.93)	8 (7.92%)	1 (0.99%)
2	7	2.47	(1.20–5.02)	6 (5.94%)	1 (0.99%)
3	6	2.12	(0.98–4.55)	6 (5.94%)	0 (0.00%)
5	7	2.47	(1.20–5.02)	7 (6.93%)	0 (0.00%)
7	4	1.41	(0.55–3.58)	4 (3.96%)	0 (0.00%)
10	2	0.71	(0.19–2.54)	2 (1.98%)	0 (0.00%)
15	0	0.00	(0.00–1.34)	0 (0.00%)	0 (0.00%)
20	0	0.00	(0.00–1.34)	0 (0.00%)	0 (0.00%)

The Wilson method was used for 95% confidence intervals. Patient percentages calculated from the total of 101 patients

### Stratification by ^18^F-FDG uptake period

Supplementary Table S3 and Supplementary Figs. S2 and S3 present the estimated marginal means and pairwise comparisons for the ≤ 80 min (*n* = 49) and > 80 min (*n* = 52) uptake period groups.

In the ≤ 80 min group, L_SUV_max,_ L_SUV_mean_, L_SUV_sd_, and LBR showed no significant differences from 1.5 min onward (all *p* ≥ 0.087). In the > 80 min group, these parameters showed no significant differences from 3 min onward (all *p* ≥ 0.160). MTV did not differ across any time points in either subgroup (all *p* = 1.000). Detailed pairwise comparisons for other metrics are provided in the Supplementary Material.

### Stratification by lesion size

Supplementary Table S4 and Supplementary Figs. S4 and S5 present the estimated marginal means and pairwise comparisons for the < 15 mm (*n* = 96), 15–20 mm (*n* = 81), and > 20 mm (*n* = 106) lesion size groups.

For lesions < 15 mm, L_SUV_max_, L_SUV_mean_, L_SUV_sd_, and LBR showed no significant differences from 3 min onward (all *p* = 1.000), and MTV showed no differences across any time points (all *p* = 1.000). For lesions 15–20 mm and > 20 mm, these parameters showed no significant differences from 2 min onward (all *p* = 1.000), and MTV also showed no differences across any time points (all *p* = 1.000).

## Discussion

In this retrospective simulation study, the minimal PET acquisition time required to match a 20-min reference depends on the clinical goal. Quantitative parameters (L_SUV_max_, L_SUV_mean_, L_SUVsd, LBR) stabilized from 3 min onward, subjective image quality plateaued at 7 min, and INR required 10 min. These findings provide evidence-based guidance for tailoring scan duration to specific diagnostic tasks.

Among these quantitative parameters, the negative correlation between L_SUV_max_ and acquisition time deserves attention. Although longer acquisitions improve image quality, L_SUV_max_ paradoxically decreased. This phenomenon is explained by noise-induced bias. Longer acquisitions reduce noise, mitigating SUV_max_ inflation [22]. Conversely, shorter acquisitions artificially elevate L_SUV_max_ [23, 24]. As acquisition time extends, noise is reduced and bias is eliminated. Consequently, the decrease in L_SUV_max_ reflects improved accuracy, not deteriorated quantitation. This relationship has been consistently reported in PET/MRI studies [10, 25]. Our data confirm that bias is eliminated by 3 min, after which L_SUV_max_ remains stable.

In contrast to the early stabilization of quantitative parameters, subjective image quality plateaued later. Scores at 5 min remained significantly lower than at 20 min, whereas from 7 min onward, no differences were observed. This delay likely reflects the higher SNR required for human observers. Visual detection in noisy PET images depends critically on SNR. As acquisition time increases, noise is reduced and observer performance improves until a plateau [26]. Previous studies found similarly that subjective quality declines at short acquisitions and stabilizes after a minimum duration [12, 27]. Thus, 7 min is the earliest time point at which subjective quality matches the full protocol, supporting its use for routine diagnostic imaging. The reliability of these subjective assessments is further supported by inter‑observer agreement analysis. Weighted kappa values improved from substantial at shorter acquisition times to almost perfect at longer times. At 7 min, the kappa value was 0.751, indicating good agreement between readers. The lower agreement at very short acquisitions reflects increased image noise, but such extreme scan durations are not clinically relevant. The substantial agreement at 7 min thus further reinforces the 7 min recommendation.

Our subgroup analysis revealed that patients with a shorter ¹⁸F-FDG uptake period (≤ 80 min) or larger lesions (≥ 15 mm) achieved stable SUV parameters as early as 2 min, whereas those with a longer uptake period (> 80 min) or smaller lesions (< 15 mm) required 3 min. This finding is physiologically plausible. A shorter uptake period likely occurs during the phase of rapid ¹⁸F-FDG accumulation in tumors, where image contrast is already high and signal-to-noise ratio improves quickly with longer acquisition times [28]. Conversely, with longer uptake periods, ¹⁸F-FDG clearance may have begun, and further extending the scan duration yields diminishing returns in noise reduction, thus requiring more time to match the reference standard [29]. For lesion size, smaller lesions are more susceptible to partial volume effects and noise, necessitating longer acquisitions to achieve SUV stability [30]. These observations underscore the potential for individualizing PET acquisition protocols based on patient- and lesion-specific factors.

Lesion detectability further supports the 7 min protocol. The miss rate at 7 min was low (1.4%, 95% CI 0.6–3.6%), and only 4% of patients had any missed lesions (none missed two or more). In contrast, at 5 min, the miss rate nearly doubled to 2.5% and affected 7% of patients. Missed lesions at 5–10 min were predominantly small nodules near the PET resolution limit, a finding consistent with the known limitations of partial volume effects and count statistics for sub-centimeter lesions [30]. At very short acquisitions (0.5–3 min), high background noise was the dominant masking factor, reflecting the insufficient signal-to-noise ratio for lesion detection [31]. These observations align with our quantitative and subjective findings: small lesions and high noise both delay the achievement of stable image quality. Collectively, these data confirm the clinical safety of a 7 min acquisition, as the miss rate and patient-level impact are minimal, and the causes of missed lesions are well understood.

Our findings align with previous studies that demonstrated the feasibility of reducing PET acquisition times. Murthy et al reported that 7 min acquisition improved image quality in ⁶⁸Ga-PSMA-11 PET/MRI for prostate cancer [10]. Jannusch et al found that 5 min was sufficient for breast cancer staging with ¹⁸F-FDG PET/MRI [31], and Lindemann et al reduced whole-body PET/MRI to 2 min per bed position [32]. Building on these observations, the present study provides several methodological advances. By applying linear mixed-effects models that account for the hierarchical structure of lesion-level data, and by performing subgroup analyses based on ¹⁸F-FDG uptake period and lesion size, we offer more robust estimates and individualized time recommendations than previously available.

Our results have direct implications for clinical practice. For routine diagnostic imaging, a 7-min acquisition is recommended, as it provides the earliest plateau in subjective quality and a low miss rate. For quantitative assessments, 3–5 min suffices, which could increase scanner throughput and reduce motion artifacts. Patients with shorter ¹⁸F-FDG uptake periods or larger lesions might tolerate even 2-min acquisitions, enabling individualized protocols.

Several limitations should be acknowledged. First, the retrospective, single-center, single-scanner (GE Healthcare Signa) design limits generalizability to other platforms (*e.g*., Siemens Healthineers, Philips Healthcare). However, the fundamental physical principles suggest that similar trends should apply. Second, the data‑driven thresholds (80-min uptake period, lesion size) and the use of a 20-min reference (not an external gold standard) require validation. Third, the small number of missed lesions precluded robust correlation analyses, and their descriptive characterization lacked quantitative thresholds. Fourth, only standard tracer activity and a 20-min reference were tested. Other dose regimens or longer acquisitions need further study. Prospective multicenter validation is warranted.

In conclusion, the minimal PET acquisition time for chest ¹⁸F‑FDG PET/MRI depends on the clinical objective. A 7-min acquisition provides subjective image quality and lesion detectability comparable to a 20-min protocol, making it suitable for routine diagnostic imaging. For quantitative tasks, 3–5 min is sufficient, and patients with shorter ¹⁸F-FDG uptake periods or larger lesions may tolerate even 2 min. Adoption of a 7-min protocol can improve patient comfort and increase scanner throughput. Prospective multicenter studies are warranted to validate these time thresholds and explore further individualization.

## Supplementary information

**Additional File 1: Figure S1.** Scatter plot of lesion size *versus* first detectable acquisition time. **Figure S2.** Quantitative PET parameters across PET acquisition times stratified by 18F-FDG uptake period. **Figure S3.** Heatmaps of pairwise comparison p-values for quantitative PET parameters stratified by 18F-FDG uptake period. **Figure S4.** Quantitative PET parameters across PET acquisition times stratified by lesion size. **Figure S5.** Heatmaps of pairwise comparison p-values for quantitative PET parameters stratified by lesion size. **Table S1.** Patient and lesion characteristics (n = 101). **Table S2.** Detailed characteristics of missed lesions. **Table S3.** Estimated marginal means of quantitative PET parameters stratified by 18F-FDG uptake period (≤ 80 min vs. > 80 min). **Table S4.** Estimated marginal means (95% CI) of quantitative PET parameters stratified by lesion size (< 15 mm, 15–20 mm, and > 20 mm).

## Data Availability

The datasets used and analyzed in this study are available from the corresponding author upon reasonable request.

## Abbreviations

^18^F-FDG: ^18^F-fluorodeoxyglucose
CT: Computed tomography
INR: Image noise ratio
L_SNR: Signal-to-noise ratio of the lesion
L_SUV_max_: Maximum standardized uptake value of the lesion
L_SUV_mean_: Mean standardized uptake value of the lesion
L_SUV_sd_: Standard deviation of lesion standardized uptake value
LBR: Lesion-to-background ratio
MRI: Magnetic resonance imaging
MTV: Metabolic tumor volume
PET: Positron emission tomography
PSMA: Prostate-specific membrane antigen
SNR: Signal-to-noise ratio
SUV: Standardized uptake value
