# Supplementary material for: Optimization of PET acquisition time for thoracic lesions in 18F-FDG PET/MRI: a retrospective analysis
Source: Eur Radiol Exp. 2026 Oct 7;10:151. doi: 10.1186/s41747-026-00787-y (PMC13646212; doi:10.1186/s41747-026-00787-y)
Supplement: Supplementary file 1 [file 41747_2026_787_MOESM1_ESM.pdf]

Optimization of PET acquisition time for thoracic lesions in 18F-FDG PET/MRI: a retrospective analysis

ELECTRONIC SUPPLEMENTARY MATERIAL

Eur Radiol Exp (2026) Gu H, Jiang Y, Cai J et al.

Supplementary Table S1. Patient and lesion characteristics (n = 101)

Parameter	Characteristic	Value
Demographics	Age (years), mean ± SD	63.8 ± 9.6
	Sex (male / female)	77 / 24
Primary diagnosis	– Lung cancer (total)	80
	• Adenocarcinoma	35
	• Squamous cell carcinoma	14
	• Small cell carcinoma	9
	• Other or unspecified*	22
	– Esophageal cancer	15
	– Other thoracic malignancies**	6
Diagnostic confirmation	–Pathologically confirmed	89 (88.1%)
	– Image-only (no pathology)	12 (11.9%)
Disease status	– Post-operative examination	30 (29.7%)
	– Presence of distant metastasis	55 (54.5%)
	– Presence of lymph node metastasis	60 (59.4%)
Lesion size (spherical diameter)	– < 15 mm	96
	– 15–20 mm	81
	– > 20 mm	106

Data are presented as mean ± standard deviation (SD) or counts (with percentages where applicable).
*Includes high-grade neuroendocrine carcinoma (n = 1), non-small cell carcinoma (n = 1), image-only diagnosis without pathology (n = 7), and other lung cancer types or unclassified (n = 13).
**Includes lymphoma (n = 2), thymic carcinoma (n = 1), multiple myeloma (n = 1), gastric cancer metastasis (n = 1), nasopharyngeal carcinoma metastasis (n = 1).

Supplementary Table S2. Detailed characteristics of missed lesions

Lesion ID	First detectable time (min)	Size (mm)	MTV	L_SUV_{max}	IN	Location	Diagnosis
7_2	1	16.57	2.38	8.65	0.10	Right lower lobe	Invasive adenocarcinoma of the lung
8_1	10	13.47	1.28	4.10	0.05	Right upper lobe	Invasive adenocarcinoma of the lung
18_1	1.5	19.45	3.8	3.98	0.13	Middle mediastinum	Pulmonary inflammation
20_2	3	22.98	6.35	4.59	0.08	Right upper lobe	Postoperative lung cancer with bone metastasis
20_3	3	28.53	12.16	5.63	0.08	Left lower lobe	Postoperative lung cancer with bone metastasis
21_2	1.5	23.87	7.12	9.36	0.19	Right lower lobe	Lung adenocarcinoma
29_4	7	30.47	14.81	5.07	0.07	Left upper lobe	Non-small cell lung cancer
30_2	1	31.62	16.55	13.47	0.06	Left lower lobe	Lung cancer
36_2	1	10.53	0.61	2.29	0.06	Right upper lobe	B-cell non-Hodgkin lymphoma
38_4	1	15.15	1.82	5.75	0.08	Right lower lobe	Lung cancer
47_2	1	17.08	2.61	4.44	0.17	Lower mediastinum	Nasopharyngeal carcinoma with cervical metastasis
57_1	1	10.79	0.66	5.61	0.14	Right lower lobe	Invasive adenocarcinoma of the lung
60_2	1	20.93	4.80	5.46	0.15	Middle mediastinum	Esophageal cancer
65_1	1	14.04	1.45	5.01	0.12	Right upper lobe	Peripheral lung cancer
71_1	0.5	12.12	0.93	4.33	0.06	Right upper lobe	Small cell lung cancer
72_2	1	22.39	5.88	4.78	0.25	Lower mediastinum	Lung adenocarcinoma
84_4	1	13.15	1.19	3.40	0.07	Left lower lobe	Invasive adenocarcinoma of the lung
89_5	1	13.37	1.25	11.85	0.11	Right middle lobe	Poorly differentiated lung adenocarcinoma
92_1	7	11.98	0.90	8.23	0.07	Upper thoracic esophagus	Esophageal squamous cell carcinoma
93_6	7	14.42	1.57	3.94	0.09	Mediastinum (Thymus)	Thymic squamous cell carcinoma
94_2	2	13.26	1.22	4.59	0.10	Upper mediastinum	Postoperative esophageal cancer
96_5	1.5	15.34	1.89	7.72	0.08	Right lower lobe	Lung cancer
97_2	1	13.85	1.39	3.07	0.04	Left upper lobe	Invasive adenocarcinoma of the lung
99_3	7	18.94	3.56	4.97	0.04	Right lower lobe	Postoperative lung adenocarcinoma

All parameters were measured at the 20 min reference group. First detectable time indicates the shortest PET acquisition time at which the lesion was identified. Lesion size (mm) was derived from MTV as equivalent spherical diameter.

L_SUV_{max} Maximum standardized uptake value of the lesion, *IN* Image noise ratio, *MTV* Metabolic tumor volume (cm^3)

Supplementary Table S3. Estimated marginal means of quantitative PET parameters stratified by ^{18}F-FDG uptake period (≤ 80 min vs. > 80 min)

Metric	Group	0.5 min	1 min	1.5 min	2 min	3 min	5 min	7 min	10 min	15 min	20 min
L_SUV_{max}	≤ 80 min	9.52 (8.70–10.34)	8.60 (7.78–9.42)	8.40 (7.58–9.22)	8.24 (7.42–9.06)	8.12 (7.30–8.94)	7.93 (7.11–8.75)	7.90 (7.08–8.72)	7.95 (7.14–8.77)	7.99 (7.17–8.81)	8.06 (7.24–8.87)
	> 80 min	10.13 (9.31–10.94)	9.03 (8.22–9.84)	8.99 (8.18–9.81)	8.70 (7.89–9.51)	8.37 (7.56–9.18)	7.98 (7.17–8.80)	7.99 (7.18–8.80)	7.98 (7.17–8.79)	8.02 (7.21–8.83)	8.02 (7.20–8.83)
L_SUV_{mean}	≤ 80 min	4.61 (4.23–4.99)	4.35 (3.97–4.73)	4.26 (3.87–4.64)	4.17 (3.78–4.55)	4.11 (3.73–4.49)	4.02 (3.64–4.40)	4.03 (3.65–4.42)	4.06 (3.68–4.45)	4.07 (3.69–4.46)	4.09 (3.71–4.48)
	> 80 min	4.92 (4.49–5.36)	4.55 (4.11–4.98)	4.56 (4.12–4.99)	4.42 (3.99–4.86)	4.28 (3.85–4.71)	4.12 (3.69–4.55)	4.14 (3.70–4.57)	4.14 (3.71–4.58)	4.17 (3.73–4.60)	4.18 (3.75–4.62)
L_SUV_{sd}	≤ 80 min	1.43 (1.30–1.57)	1.33 (1.19–1.47)	1.29 (1.16–1.43)	1.27 (1.13–1.41)	1.26 (1.12–1.40)	1.22 (1.09–1.36)	1.23 (1.09–1.36)	1.24 (1.11–1.38)	1.25 (1.11–1.39)	1.26 (1.12–1.40)
	> 80 min	1.58 (1.44–1.72)	1.42 (1.28–1.56)	1.42 (1.28–1.56)	1.37 (1.23–1.51)	1.32 (1.19–1.46)	1.26 (1.12–1.40)	1.27 (1.13–1.41)	1.27 (1.13–1.41)	1.28 (1.14–1.42)	1.28 (1.14–1.42)
L_SNR	≤ 80 min	3.34 (3.21–3.47)	3.41 (3.28–3.54)	3.43 (3.30–3.56)	3.41 (3.29–3.54)	3.41 (3.28–3.54)	3.43 (3.30–3.56)	3.45 (3.32–3.58)	3.41 (3.28–3.54)	3.40 (3.27–3.53)	3.37 (3.24–3.50)
	> 80 min	3.21 (3.11–3.32)	3.29 (3.19–3.39)	3.30 (3.20–3.40)	3.30 (3.20–3.40)	3.31 (3.21–3.41)	3.35 (3.25–3.45)	3.34 (3.24–3.44)	3.35 (3.25–3.45)	3.35 (3.25–3.45)	3.35 (3.25–3.45)
IN	≤ 80 min	0.24 (0.23–0.26)	0.19 (0.17–0.20)	0.17 (0.15–0.18)	0.15 (0.14–0.17)	0.14 (0.12–0.15)	0.12 (0.10–0.13)	0.10 (0.09–0.12)	0.10 (0.08–0.11)	0.09 (0.07–0.10)	0.09 (0.07–0.10)
	> 80 min	0.27 (0.26–0.29)	0.21 (0.20–0.22)	0.18 (0.17–0.19)	0.17 (0.16–0.18)	0.15 (0.14–0.16)	0.13 (0.12–0.14)	0.12 (0.11–0.13)	0.12 (0.11–0.13)	0.11 (0.10–0.12)	0.11 (0.10–0.12)
LBR	≤ 80 min	17.98 (16.21–19.75)	15.81 (14.05–17.57)	15.36 (13.60–17.12)	14.91 (13.15–16.67)	14.57 (12.81–16.34)	14.23 (12.47–15.99)	14.24 (12.48–16.00)	14.30 (12.54–16.06)	14.23 (12.47–15.99)	14.25 (12.49–16.01)
	> 80 min	16.29 (15.08–17.51)	14.29 (13.08–15.50)	14.40 (13.19–15.61)	14.06 (12.85–15.27)	13.62 (12.41–14.83)	13.01 (11.80–14.22)	13.06 (11.85–14.27)	12.96 (11.75–14.17)	12.98 (11.77–14.19)	12.89 (11.68–14.10)
MTV	≤ 80 min	12.51 (5.80–19.22)	12.06 (5.35–18.77)	12.34 (5.62–19.05)	12.08 (5.37–18.79)	12.00 (5.29–18.71)	12.23 (5.52–18.94)	12.43 (5.72–19.14)	12.50 (5.79–19.21)	12.64 (5.93–19.35)	12.67 (5.96–19.38)
	> 80 min	7.37 (4.84–9.90)	7.29 (4.76–9.82)	7.49 (4.96–10.02)	7.34 (4.81–9.87)	7.24 (4.71–9.77)	7.49 (4.96–10.02)	7.44 (4.91–9.97)	7.43 (4.90–9.96)	7.46 (4.93–9.99)	7.47 (4.94–10.00)

Data are presented as estimated marginal means (95% confidence interval). All metrics were analyzed using linear mixed-effects models with acquisition time as a fixed effect and random intercepts for patients and for lesions nested.

L_SUV_{max} Maximum standardized uptake value of the lesion, *L_SUV_{mean}* Mean standardized uptake value of the lesion, *L_SUV_{sd}* Standard deviation of standardized uptake value within the lesion, *L_SNR* Lesion signal-to-noise ratio, *IN* Image noise ratio, *LBR* Lesion-to-background ratio, *MTV* Metabolic tumor volume (cm³)

Supplementary Table S4. Estimated marginal means (95% CI) of quantitative PET parameters stratified by lesion size (< 15 mm, 15–20 mm, and > 20 mm)

Metric	Group	0.5 min	1 min	1.5 min	2 min	3 min	5 min	7 min	10 min	15 min	20 min
L_SUV$_{max}$	< 15 mm	8.40 (7.69–9.12)	7.54 (6.83–8.25)	7.33 (6.62–8.04)	7.08 (6.37–7.79)	6.74 (6.03–7.45)	6.44 (5.73–7.15)	6.42 (5.71–7.12)	6.39 (5.68–7.09)	6.43 (5.73–7.14)	6.45 (5.74–7.16)
	15–20 mm	9.52 (8.74–10.30)	8.34 (7.56–9.12)	8.24 (7.46–9.01)	7.85 (7.07–8.63)	7.64 (6.86–8.42)	7.39 (6.62–8.17)	7.43 (6.65–8.21)	7.45 (6.67–8.23)	7.44 (6.66–8.22)	7.46 (6.68–8.23)
	> 20 mm	11.33 (10.47–12.20)	10.30 (9.43–11.17)	10.27 (9.40–11.14)	10.17 (9.30–11.03)	10.01 (9.14–10.87)	9.66 (8.79–10.53)	9.64 (8.77–10.50)	9.69 (8.82–10.55)	9.76 (8.89–10.63)	9.80 (8.94–10.67)
L_SUV$_{mean}$	< 15 mm	4.42 (4.05–4.78)	4.01 (3.65–4.37)	3.90 (3.54–4.26)	3.78 (3.42–4.14)	3.59 (3.23–3.94)	3.42 (3.06–3.78)	3.43 (3.07–3.79)	3.42 (3.06–3.78)	3.45 (3.09–3.81)	3.47 (3.11–3.83)
	15–20 mm	4.78 (4.39–5.17)	4.25 (3.86–4.64)	4.23 (3.84–4.62)	4.01 (3.62–4.40)	3.93 (3.54–4.32)	3.81 (3.43–4.20)	3.83 (3.44–4.22)	3.85 (3.46–4.24)	3.83 (3.44–4.22)	3.82 (3.44–4.21)
	> 20 mm	5.07 (4.61–5.52)	4.95 (4.50–5.41)	4.97 (4.52–5.42)	4.95 (4.50–5.40)	4.91 (4.46–5.36)	4.80 (4.34–5.25)	4.82 (4.37–5.27)	4.85 (4.40–5.31)	4.89 (4.44–5.35)	4.93 (4.47–5.38)
L_SUV$_{sd}$	< 15 mm	1.39 (1.26–1.52)	1.24 (1.11–1.37)	1.21 (1.08–1.34)	1.17 (1.04–1.30)	1.12 (0.99–1.25)	1.06 (0.93–1.19)	1.05 (0.92–1.19)	1.05 (0.92–1.18)	1.06 (0.93–1.19)	1.07 (0.94–1.20)
	15–20 mm	1.52 (1.37–1.66)	1.33 (1.19–1.47)	1.33 (1.19–1.47)	1.26 (1.12–1.40)	1.23 (1.09–1.37)	1.18 (1.04–1.32)	1.19 (1.05–1.33)	1.20 (1.06–1.34)	1.20 (1.06–1.34)	1.20 (1.06–1.34)
	> 20 mm	1.61 (1.47–1.75)	1.52 (1.37–1.66)	1.51 (1.37–1.65)	1.50 (1.36–1.64)	1.49 (1.35–1.63)	1.44 (1.30–1.59)	1.45 (1.31–1.59)	1.47 (1.33–1.62)	1.49 (1.35–1.63)	1.49 (1.35–1.63)
L_SNR	< 15 mm	3.33 (3.18–3.47)	3.37 (3.22–3.51)	3.36 (3.22–3.51)	3.36 (3.21–3.50)	3.35 (3.21–3.50)	3.38 (3.23–3.52)	3.40 (3.25–3.55)	3.40 (3.25–3.55)	3.40 (3.25–3.55)	3.40 (3.25–3.55)
	15–20 mm	3.24 (3.12–3.36)	3.28 (3.16–3.41)	3.31 (3.19–3.44)	3.30 (3.18–3.42)	3.33 (3.21–3.45)	3.36 (3.24–3.48)	3.35 (3.22–3.47)	3.33 (3.21–3.45)	3.32 (3.20–3.44)	3.30 (3.18–3.42)
	> 20 mm	3.24 (3.11–3.36)	3.36 (3.23–3.50)	3.30 (3.16–3.43)	3.30 (3.16–3.43)	3.31 (3.17–3.44)	3.35 (3.21–3.48)	3.34 (3.21–3.48)	3.35 (3.21–3.48)	3.35 (3.21–3.48)	3.35 (3.21–3.48)
IN	< 15 mm	0.25 (0.24–0.26)	0.19 (0.18–0.20)	0.16 (0.15–0.17)	0.15 (0.14–0.16)	0.14 (0.12–0.15)	0.11 (0.10–0.12)	0.11 (0.10–0.12)	0.10 (0.09–0.11)	0.09 (0.08–0.10)	0.09 (0.08–0.10)
	15–20 mm	0.25 (0.24–0.27)	0.20 (0.19–0.21)	0.17 (0.16–0.18)	0.16 (0.15–0.17)	0.15 (0.14–0.16)	0.13 (0.12–0.14)	0.11 (0.11–0.12)	0.11 (0.10–0.11)	0.09 (0.09–0.10)	0.09 (0.09–0.10)
	> 20 mm	0.27 (0.26–0.28)	0.20 (0.19–0.21)	0.18 (0.17–0.19)	0.16 (0.15–0.17)	0.15 (0.14–0.16)	0.13 (0.12–0.14)	0.11 (0.11–0.12)	0.11 (0.10–0.11)	0.10 (0.10–0.11)	0.10 (0.10–0.11)
LBR	< 15 mm	14.37 (13.02–15.71)	12.99 (11.65–14.32)	12.59 (11.25–13.92)	12.20 (10.87–13.53)	11.65 (10.32–12.99)	11.17 (9.84–12.51)	11.22 (9.88–12.55)	11.07 (9.74–12.40)	11.03 (9.70–12.36)	11.02 (9.69–12.35)
	15–20 mm	16.08 (14.64–17.51)	13.81 (12.37–15.24)	13.54 (12.11–14.96)	12.98 (11.55–14.40)	12.66 (11.23–14.09)	12.36 (10.93–13.78)	12.45 (11.02–13.88)	12.45 (11.02–13.88)	12.41 (10.98–13.84)	12.30 (10.87–13.73)
	> 20 mm	19.82 (18.26–21.38)	17.28 (15.73–18.84)	17.48 (15.92–19.04)	17.22 (15.66–18.78)	16.90 (15.35–18.46)	16.26 (14.70–17.81)	16.22 (14.67–17.78)	16.28 (14.72–17.83)	16.30 (14.74–17.86)	16.28 (14.72–17.83)
MTV	< 15 mm	1.23 (1.08–1.37)	1.10 (0.97–1.24)	1.07 (0.94–1.21)	1.08 (0.95–1.22)	1.07 (0.93–1.21)	1.12 (0.99–1.26)	1.09 (0.96–1.23)	1.13 (1.00–1.27)	1.11 (0.97–1.24)	1.11 (0.97–1.24)
	15–20 mm	2.59 (2.35–2.82)	2.60 (2.37–2.83)	2.74 (2.51–2.97)	2.63 (2.40–2.86)	2.59 (2.36–2.81)	2.67 (2.44–2.90)	2.64 (2.42–2.87)	2.65 (2.42–2.87)	2.67 (2.44–2.90)	2.68 (2.45–2.91)
	> 20 mm	19.28 (13.87–24.69)	18.72 (13.31–24.13)	19.27 (13.86–24.68)	18.81 (13.40–24.21)	18.62 (13.21–24.03)	19.15 (13.74–24.56)	19.37 (13.96–24.78)	19.41 (14.00–24.81)	19.62 (14.22–25.03)	19.65 (14.24–25.06)

Data are presented as estimated marginal means (95% confidence interval). All metrics were analyzed using linear mixed-effects models with acquisition time as a fixed effect and random intercepts for patients and for lesions nested. Lesion size groups are based on MTV-derived equivalent spherical diameter. *L_SUV$_{max}$* Maximum standardized uptake value of the lesion, *L_SUV$_{mean}$* Mean standardized uptake value of the lesion, *L_SUV$_{sd}$* Standard deviation of standardized uptake value within the lesion, *L_SNR* Lesion signal-to-noise ratio, *IN* Image noise ratio, *LBR* Lesion-to-background ratio, *MTV* Metabolic tumor volume (cm^3)

Supplementary Figure S1. Scatter plot of lesion size versus first detectable acquisition time.

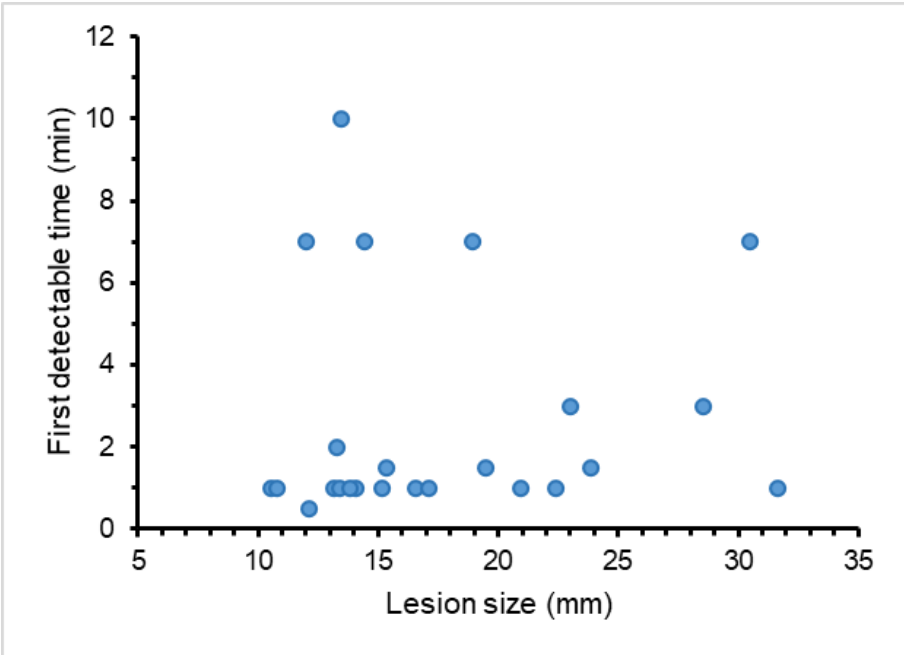

Each dot represents one lesion (n = 24). Lesion size (mm) was derived from metabolic tumor volume at the 20 min reference acquisition as equivalent spherical diameter. First detectable time indicates the shortest PET acquisition time at which the lesion was identified.

Supplementary Figure S2. Quantitative PET parameters across PET acquisition times stratified by ^{18}F-FDG uptake period

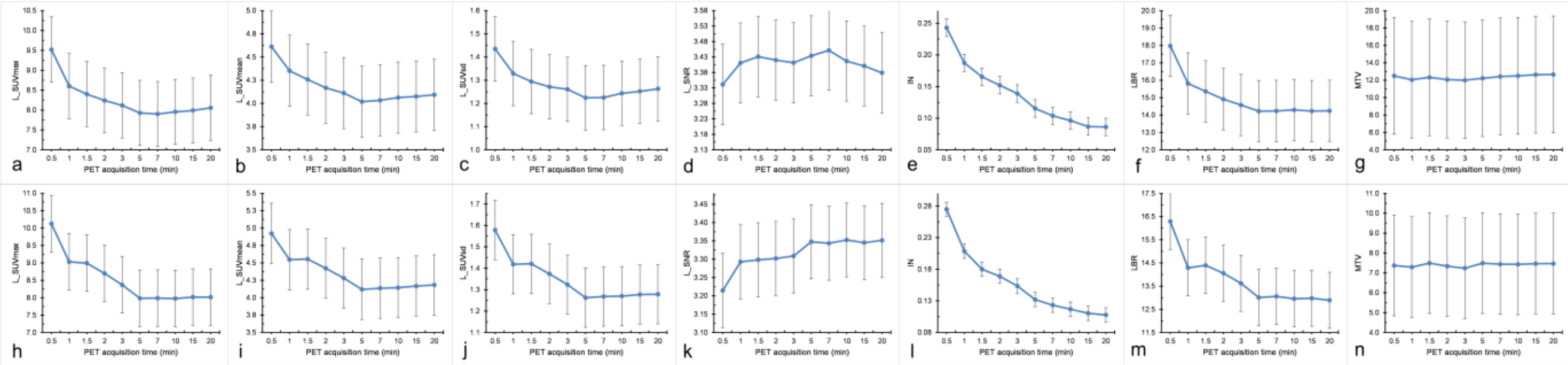

Estimated marginal means of L_SUV$_{max}$, L_SUV$_{mean}$, L_SUV$_{sd}$, L_SNR, IN, LBR, and MTV are plotted against PET acquisition time (0.5–20 min) for the ≤ 80 min uptake period group (a–g) and the > 80 min group (h–n). Error bars represent 95% CI.

L_SUV$_{max}$ Maximum standardized uptake value of the lesion, *L_SUV$_{mean}$* Mean standardized uptake value of the lesion, *L_SUV$_{sd}$* Standard deviation of standardized uptake value within the lesion, *L_SNR* Lesion signal-to-noise ratio, *IN* Image noise ratio, *LBR* Lesion-to-background ratio, *MTV* Metabolic tumor volume (cm^3)

Supplementary Figure S3. Heatmaps of pairwise comparison *p*-values for quantitative PET parameters stratified by ^{18}F-FDG uptake period

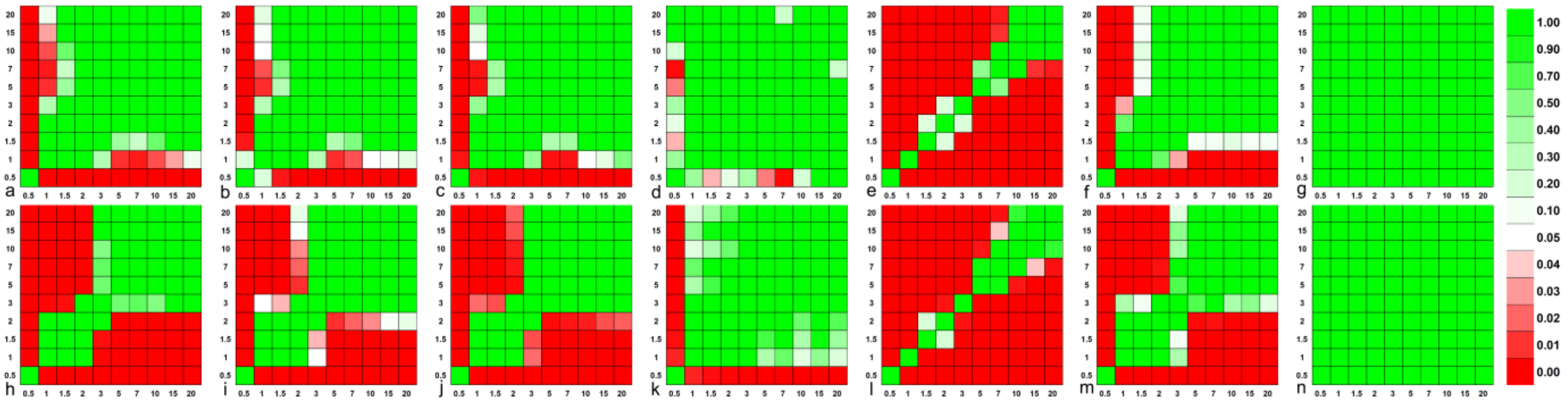

Heatmaps show Bonferroni-corrected *p*-values for all pairwise comparisons among PET acquisition times (0.5–20 min) in the ≤ 80 min uptake period group (a–g) and the > 80 min group (h–n). Each panel corresponds to one of the seven quantitative parameters: (a, h) L_SUV_{max}, (b, i) L_SUV_{mean}, (c, j) L_SUV_{sd}, (d, k) L_SNR, (e, l) IN, (f, m) LBR, (g, n) MTV. Red shades indicate significant differences ($p < 0.05$), green shades indicate non-significant differences ($p \geq 0.05$). Solid green blocks indicate stable plateaus where all comparisons among consecutive time points are non-significant.

Supplementary Figure S4. Quantitative PET parameters across PET acquisition times stratified by lesion size

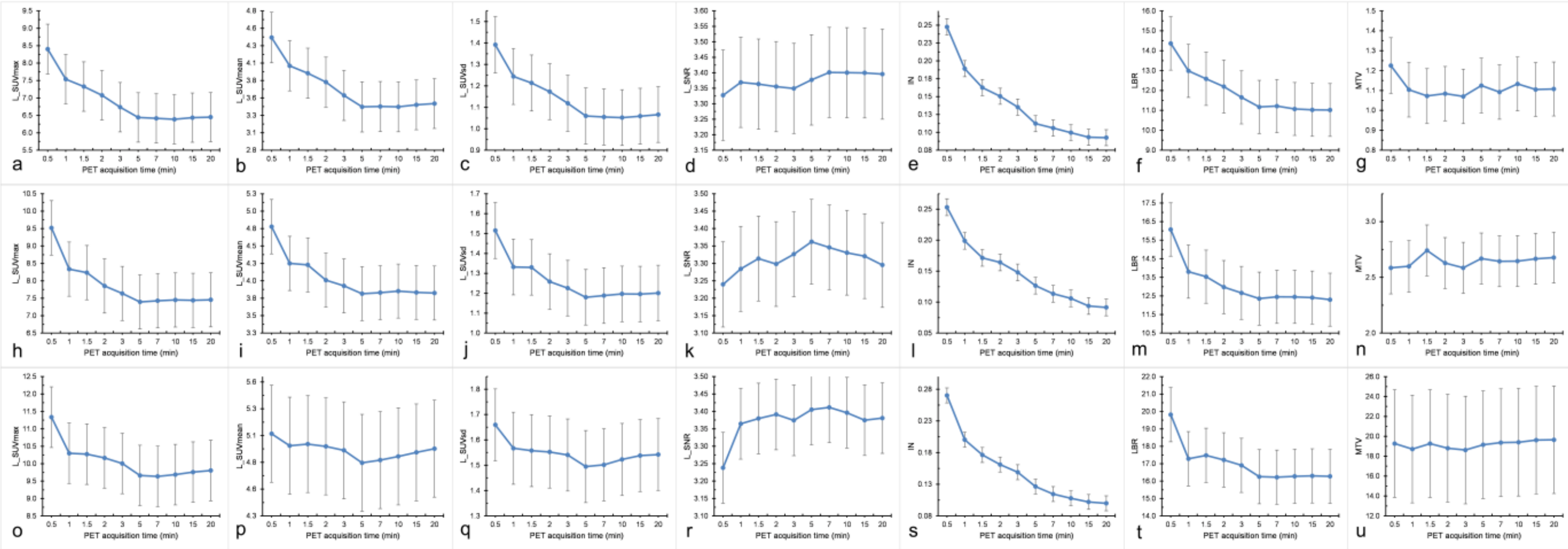

Estimated marginal means of L_SUV_{max}, L_SUV_{mean}, L_SUV_{sd}, L_SNR, IN, LBR, and MTV are plotted against PET acquisition time (0.5–20 min) for the < 15 mm group (a–g), the 15–20 mm group (h–n), and the > 20 mm group (o–u). Error bars represent 95% CI.

L_SUV_{max} Maximum standardized uptake value of the lesion, *L_SUV_{mean}* Mean standardized uptake value of the lesion, *L_SUV_{sd}* Standard deviation of standardized uptake value within the lesion, *L_SNR* Lesion signal-to-noise ratio, *IN* Image noise ratio, *LBR* Lesion-to-background ratio, *MTV* Metabolic tumor volume (cm³)

Supplementary Figure S5. Heatmaps of pairwise comparison *p*-values for quantitative PET parameters stratified by lesion size

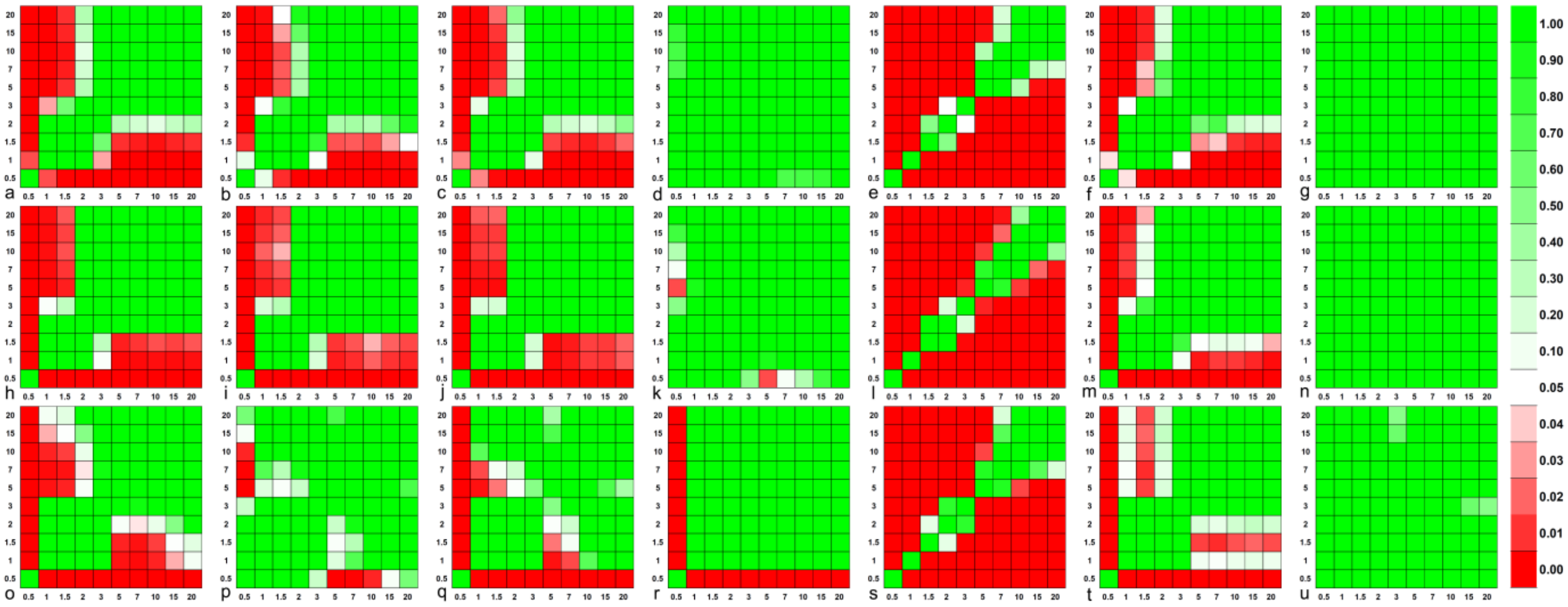

Heatmaps show Bonferroni-corrected *p*-values for all pairwise comparisons among PET acquisition times (0.5–20 min) in the three lesion size groups: < 15 mm (a–g), 15–20 mm (h–n), and > 20 mm (o–u). Each panel corresponds to one of the seven quantitative parameters: (a, h, o) L_SUV$_{max}$, (b, I, p) L_SUV$_{mean}$, (c, j, q) L_SUV$_{sd}$, (d, k, r) L_SNR, (e, l, s) IN, (f, m, t) LBR, (g, n, u) MTV. Red shades indicate significant differences ($p < 0.05$); green shades indicate non-significant differences ($p \geq 0.05$). Solid green blocks indicate stable plateaus where all comparisons among consecutive time points are non-significant.
